# Acute Pulmonary Embolism in Emergency Department Patients Despite Therapeutic Anticoagulation

**DOI:** 10.5811/westjem.2018.1.35586

**Published:** 2018-04-06

**Authors:** Michelle Y. Liu, Dustin W. Ballard, Jie Huang, Adina S. Rauchwerger, Mary E. Reed, Sean C. Bouvet, David R. Vinson

**Affiliations:** * Kaiser Permanente, Division of Research, Oakland, California; † Kaiser Permanente, San Rafael Medical Center, Department of Emergency Medicine, San Rafael, California; ‡ Kaiser Permanente, Walnut Creek Medical Center, Department of Emergency Medicine, Walnut Creek, California; § Kaiser Permanente, Sacramento Medical Center, Department of Emergency Medicine, Sacramento, California

## Abstract

**Introduction:**

Emergency department (ED) patients with acute pulmonary embolism (PE) despite therapeutic anticoagulation at the time of diagnosis are uncommonly encountered and present a diagnostic and management challenge. Their characterization and outcomes are poorly described. We sought to describe the prevalence and characteristics of therapeutically anticoagulated patients among a population of patients with acute PE in a community setting and to describe treatment changes and 30-day outcomes.

**Methods:**

From a large retrospective cohort of adults with acute, objectively-confirmed PE across 21 EDs between 01/2013 and 04/2015, we identified patients who arrived on direct oral or injectable anticoagulants, or warfarin with an initial ED international normalized ratio (INR) value ≥2.0. Patients were excluded from the larger cohort if they had received a diagnosis of venous thromboembolism (VTE) in the prior 30 days. We gathered demographic and clinical variables from electronic health records and structured manual chart review. We report discharge anticoagulation regimens and major 30-day adverse outcomes.

**Results:**

Among 2,996 PE patients, 36 (1.2%) met study criteria. Mean age was 63 years. Eleven patients (31%) had active cancer and 25 (69%) were high risk on the PE Severity Index (Classes III–V), comparable to the larger cohort (p>0.1). Reasons for pre-arrival anticoagulation were VTE treatment or prevention (n=21), and atrial fibrillation or flutter (n=15). All patients arrived on warfarin and one was also on enoxaparin: 32 had a therapeutic INR (2.0–3.0) and four had a supratherapeutic INR (>3.0). Fifteen patients (42%) had at least one subtherapeutic INR (<2.0) in the 14 days preceding their diagnostic visit. Two patients died during hospitalization. Of the 34 ultimately discharged, 22 underwent a change in anticoagulation drug or dosing, 19 of whom received injectables, either to replace or to supplement warfarin. Four patients also received inferior vena cava filters. Thirty-day outcomes included one major hemorrhage and one additional death. No patients experienced recurrent or worsening VTE.

**Conclusion:**

We found a low prevalence of therapeutic anticoagulation at the time of acute PE diagnosis. Most patients with breakthrough PE underwent a change in therapy, though management varied widely. Subtherapeutic anticoagulation levels in the preceding weeks were common and support the importance of anticoagulation adherence.

## INTRODUCTION

Acute pulmonary embolism (PE) is a common condition and is associated with significant morbidity and mortality. 1 Diagnosis of PE can be difficult, even more so in patients with suggestive signs and symptoms who are already therapeutically anticoagulated at the time of diagnosis. One study suggests that as many as 6.0% of patients diagnosed with acute PE were therapeutically anticoagulated at the time of diagnosis (what we describe as “breakthrough PE”). 2 Case reports have described patients presenting with acute venous thromboembolism (VTE) despite therapeutic international normalized ratios (INR) (2.0–3.0). 3, 4

Management of these patients poses an additional challenge as there is little consensus on treatment for breakthrough PE. The American Heart Association 2012 and European Society of Cardiology 2014 guidelines state that inferior vena cava (IVC) filters can be effective for patients with breakthrough VTE despite therapeutic anticoagulation, whereas the American College of Chest Physicians 2016 guideline recommends against IVC filter placement (Grade 1B) and instead recommends a switch from oral anticoagulants to low-molecular-weight heparin (LMWH) for at least one month (Grade 2C). 5–7 To the best of our knowledge, there is little to no evidence to guide these recommendations. Overall, better understanding of clinical characteristics, management, and outcomes of patients with breakthrough PE in a community emergency department (ED) setting is needed to inform management guidelines for these patients.

In a multicenter, retrospective, cohort study of patients with acute, objectively-confirmed PE, we sought to (1) estimate the prevalence of therapeutic anticoagulation at time of ED diagnosis, (2) characterize the patient cohort, (3) describe changes in treatment, and (4) report 30-day major adverse outcomes. We hypothesized that the prevalence of breakthrough PE was low and that it was associated with subtherapeutic anticoagulation in the two weeks preceding diagnosis. The results of this study may help inform the clinical approach to the management of this uncommon condition.

## METHODS

### Setting

Kaiser Permanente (KP) Northern California is a large, integrated healthcare delivery system that provides care to over four million members across 21 medical facilities and multiple clinics and ancillary services. KP members represent approximately 33% of the insured population in areas served and are comparable with respect to age, gender, and race/ethnicity. 8, 9 KP Northern California stores patient health records electronically using an Epic-based (Verona, WI) electronic health record (EHR), providing electronically accessible patient-level clinical data within hierarchical databases. 10,11

**Population Health Research Capsule**

What do we already know about this issue?

Some patients develop pulmonary embolism (PE) despite therapeutic anticoagulation. The prevalence, characteristics, and treatment of these patients are not well described.

What was the research question?

What is the prevalence of breakthrough PE and what treatment changes followed the diagnosis?

What was the major finding of the study?

The prevalence of breakthrough PE was low and adjustments to anticoagulation varied widely.

How does this improve population health?

A better understanding of breakthrough PE may aid clinicians in the diagnosis and management of this challenging condition.

In 2015, the 21 study EDs had an annual median census of 56,983 visits (interquartile range [IQR] 37,841–61,005), ranging from 27,977 to 121,494 visits. All emergency care was provided by residency-trained and board-certified/prepared physicians. Computed tomography pulmonary angiography and radiology department services were available 24/7, while formal compression ultrasonography and ventilation perfusion imaging were often unavailable at various hours during the night.

During the study period, no standardized acute PE management departmental policies were in place. All patients were managed at the discretion of the treating physicians. The standard KP Northern California EHR-based discharge electronic orderset for thromboembolism at the time of the study recommended warfarin with enoxaparin bridging and is described in full elsewhere. 12 All post-discharge warfarin was managed by each facility’s pharmacy-led, telephone-based anticoagulation service.

### Selection of Participants

This retrospective cohort study identified eligible patients from the Management of Acute PuLmonary Embolism (MAPLE) study database. The MAPLE study is an observational, retrospective study of adult patients (age ≥18 years) with acute, objectively-confirmed PE presenting to 21 non-rural community medical centers across KP Northern California from January 2013 to April 2015. Study patients were identified using ICD-9 codes and manual chart confirmation as described in full elsewhere. 13, 14 Consistent with other PE studies, all cases were diagnosed either by computed tomography pulmonary angiogram, ventilation-perfusion scan, or positive extremity compression ultrasound for deep vein thrombosis with concomitant PE symptoms, such as acute onset of dyspnea or chest pain. 15–17 Patients were excluded from the MAPLE study if they had been diagnosed with acute VTE in the prior 30 days or had chronic PE, were designated comfort care status in the ED, were transferred outside the KP system from the ED or left the ED against medical advice, had insignificant PE that was untreated, were younger than 18 years at the time of diagnosis, were known to be pregnant, or were non-health plan members (Figure). This study was approved by the Kaiser Permanente Northern California Institutional Review Board and obtained a waiver of informed consent.

For this study, we identified patients within the MAPLE cohort who arrived in the ED on direct oral anticoagulants (dabigatran, rivaroxaban, apixaban, or edoxaban), injectable anticoagulants (fondaparinux or LMWHs), or warfarin with an initial ED INR value ≥2.0, the lower limit of the therapeutic range (Figure). We screened for anticoagulation use electronically using the patient’s active medications list in the EHR, then undertook manual chart review for confirmation. We excluded patients who were found through chart review not to be currently taking anticoagulants. In these cases, the elevated INR was secondary to lab error or hepatic or systemic disease.

### Data Collection

The two chart abstractors – a practicing emergency physician and a research assistant – received standardized training on data collection methods and use of the electronic data collection tool, which was modified to its final form after pilot testing. The principal investigator (DRV) answered coding questions and adjudicated any differences in chart abstraction. The two abstractors reviewed each case to confirm eligibility and change in post-discharge anticoagulation management. Interrater reliability is reported using a weighted kappa statistic as well as percent agreement.

Using a combination of EHR extraction and structured manual chart review, the abstractors confirmed and collected the following variables: age; gender; indication for anticoagulation (VTE treatment or prophylaxis and atrial fibrillation or flutter); INR measurements in the 14 days preceding the index ED visit; active cancer at the time of index ED visit; PE Severity Index (PESI) score and risk class; and 30-day major adverse outcomes: major hemorrhage, recurrent or worsening VTE, and death. 18, 19

We retrospectively calculated the PESI score and risk class at time of ED disposition using definitions from the initial derivation and validation study by Aujesky et al. and a process described in an earlier MAPLE publication. 13, 18 Active cancer was defined as cancer undergoing treatment in the prior 12 months or receiving palliative cancer care at the time of the index ED visit. Non-melanoma skin cancers were excluded. Any INR value <2.0 in the 14 days preceding the index ED visit was considered subtherapeutic.

### Outcomes

Our primary outcome was an adjustment in PE treatment for patients discharged from the ED or inpatient units. Changes included alterations in anticoagulation drug or dosing and placement of an IVC filter.

Secondary outcomes included 30-day major adverse events associated with VTE and its treatment: major hemorrhage, new or recurrent VTE, and all-cause mortality. Major hemorrhage, as defined by the International Society on Thrombosis and Haemostasis, included bleeding at high-risk anatomic locations (intracranial, intraspinal, intraocular, retroperitoneal, intra-articular, pericardial, or intramuscular with compartment syndrome), or overt bleeding with either a reduction of hemoglobin ≥2 g/dL or a transfusion of two or more units of packed red blood cells. 19 Recurrent VTE was defined as a new or expanded abnormality on imaging in a symptomatic patient. Deaths were identified using a healthcare system mortality database that links to the Social Security death master file and the California State Department of Vital Statistics to identify both in-system and out-of-system deaths. We also identified claims for out-of-system medical encounters in order to improve capture of heathcare visits related to our 30-day outcomes.

### Statistical Analysis

Means and frequencies are presented using descriptive statistics. We compared active cancer and high-risk designation on the PESI in our cohort to the larger MAPLE cohort using a chi square test with significance level of p<0.05. All analyses were conducted using GraphPad Software (La Jolla, CA).

## RESULTS

### Study Population

Of the 2,996 encounters within the MAPLE cohort, we identified electronically 46 patients as potential study candidates (Figure). After structured manual chart review, 10 were excluded for no current use of anticoagulant medication, leaving 36 patients (1.2%) who met study criteria (Figure). The two investigators agreed on 94% of the post-discharge changes in anticoagulation drug or dosing. The kappa value for the 30-day adverse outcomes ranged from 0.66 to 1.00. The percent agreement for each of the variables ranged from 97.8% to 100%, median 100% (IQR 98.9% to 100%).

### Characteristics

The mean age of the cohort was 63 years, and 25 patients (69%) were male. All patients arrived on warfarin and one was also on enoxaparin: 32 had a therapeutic INR (2.0–3.0) and four had a supratherapeutic INR (>3.0). The majority of patients were anticoagulated for VTE treatment and prevention (Table 1). Within 14 days prior to their index ED visit, 16 patients (44%) had one or more INR levels drawn and 15 patients (42%) had at least one subtherapeutic INR (<2.0) measurement with a mean INR of 1.5 (IQR 1.2–1.8), ranging from 1.0 to 1.9. Eleven patients (31%) had active cancer and 25 (69%) had higher risk PESI scores (Classes III–V), rates comparable to the larger cohort (p>0.1) (Table 1). Two patients died during hospitalization.

### Primary and Secondary Outcomes

Of the 34 patients ultimately discharged, 22 (65%) underwent a change in anticoagulation drug or dosing (Table 2). Twelve patients received no change to their existing warfarin regimen upon discharge, nine of whom had a subtherapeutic INR in the preceding 14 days. Overall, 30-day adverse outcomes included one major hemorrhage and one additional death. Of the three deaths total, two were from lung cancer and one was from bilateral PE. No patients experienced recurrent or worsening VTE.

### Patients with Subtherapeutic INR Measurements 14 Days Prior to Presentation

The mean age of the 15 patients with at least one subtherapeutic INR (<2.0) in the 14 days prior to ED presentation was 67 years. Other characteristics and demographics are described in Table 3. Patients with a subtherapeutic INR measurement in the prior 14 days were more likely to be discharged with no treatment change compared to patients without subtherapeutic INR measurements (60% vs. 16%, p<0.01).

## DISCUSSION

In this retrospective cohort study, we found a low prevalence of breakthrough PE (1.2%; 36/2,996). The majority of patients underwent a change in anticoagulant drug or dosing, with almost half replacing warfarin with injectable anticoagulants, and few (5.9%; 2/34) experienced adverse outcomes in the 30 days following discharge. Many patients had at least one subtherapeutic INR measurement in the 14 days prior to index ED visit.

Little research attention has been directed to the study of breakthrough PE. Few studies have characterized the prevalence of this condition. In a retrospective cohort study from a single, tertiary-care center in Australia, Moutzouris et al. identified 56 of 923 patients (6.1%) with acute PE who had had a therapeutic INR at the time of diagnosis. 2 Many dissimilarities between the Australian PE population and our own may account for the difference in prevalence between their study and ours (6.1% vs 1.2%). Notable among these is that the MAPLE cohort excluded patients with a recent VTE diagnosis in the preceding 30 days, thus excluding from the study those patients who may have developed breakthrough PE early in their course of treatment (that is, within the first month).

Potential contributing etiologies of breakthrough VTE include subtherapeutic anticoagulation (often attributed to suboptimal adherence), antiphospholipid syndrome, established myeloproliferative neoplasm, JAK2 V617F mutation in the absence of an established myeloproliferative neoplasm, and cancer. 20–24 In this study, we were able to assess only the predictive risk associated with subtherapeutic anticoagulation and active cancer. The high number of patients with subtherapeutic INR measurements may support prior findings of suboptimal medication adherence as a potential etiology for breakthrough VTE. 20 Prevalence of active cancer in our cohort was comparable to the larger MAPLE cohort, a finding consistent with Moutzouris et al. 2 While we were unable to substantiate prior findings suggesting active cancer as an independent predictor of recurrent PE in anticoagulated patients, our study was not designed, a priori, to test this association and we may have been underpowered to detect such a link. 4 At present, there is insufficient research on breakthrough PE to provide evidence-based guidance for the practicing clinician assessing a therapeutically anticoagulated patient with symptoms suggesting acute PE.

The changes in PE management we observed in this study are consistent with existing guidelines recommending a switch from warfarin to injectable anticoagulants or the placement of IVC filters in patients with breakthrough PE. 5–7 The majority of our patients were discontinued from warfarin and switched to injectable anticoagulants; however, among patients with at least one subtherapeutic INR in the 14 days preceding presentation, the majority received no treatment change, simply a reinforcement of prescribed dosing. The 60% of subtherapeutic patients discharged with no treatment change was significantly higher than the 14% of therapeutic patients whose treatment was unchanged. A history of pre-arrival subtherapeutic INR may guide physicians to attribute breakthrough PE to sub-optimal medication or dietary adherence, or need for long-term dosing adjustment. Although the majority of our patients were discontinued from warfarin, five patients were prescribed dual therapy of enoxaparin and warfarin, a management regimen not studied in the literature or discussed in the guidelines.

Warfarin was the oral anticoagulant of choice for the treatment of acute PE during the study period. It has since been replaced by direct oral anticoagulants as the drugs-of-choice for most patients with PE. 7 Early research suggests that adherence to the newer agents may be similar to adherence to warfarin. 25, 26 This implies that missing doses of direct oral anticoagulants may subject patients to the risk of breakthrough PE, just as with missing doses of warfarin. The half-lives of the direct oral anticoagulants are significantly shorter than that of warfarin (6–17 hours vs. 20–60 hours), suggesting less tolerance for non-adherence. 27 However, missing doses of direct oral anticoagulants may not carry greater risks than missing doses of warfarin. One small study found that only 1% of patients (2/190) developed recurrent VTE in the 30 days following several days without direct anticoagulation.^28^ Much larger studies are needed, however, to more precisely define the risk of reduced adherence.

## LIMITATIONS

Our study is limited by its retrospective nature and small study size, reducing our ability to identify significant trends within our population. We were also unable to determine the broader prevalence of breakthrough PE in the larger anticoagulated PE population as our study cohort did not include patients whose breakthrough PE went undetected or those with early breakthrough PE. We were not able to collect data on potential predictors of breakthrough PE beyond active cancer diagnosis as testing for antiphospholipid syndrome, established myeloproliferative neoplasm, and JAK2 V617F mutation are not routine in our system. We conducted this study before KP Northern California EDs switched to direct oral anticoagulant use, and thus cannot speak to this new treatment regimen. Finally, although conducted in 21 community hospitals, characteristics and results found in this study may not be generalizable to other practice settings and geographic locations.

## CONCLUSION

We found a low prevalence of breakthrough PE, few adverse 30-day outcomes, and frequent and varied changes in treatment among patients with breakthrough PE. Subtherapeutic anticoagulation levels in the preceding weeks were common, supporting the importance of anticoagulation adherence.

## Figures and Tables

**Figure:**
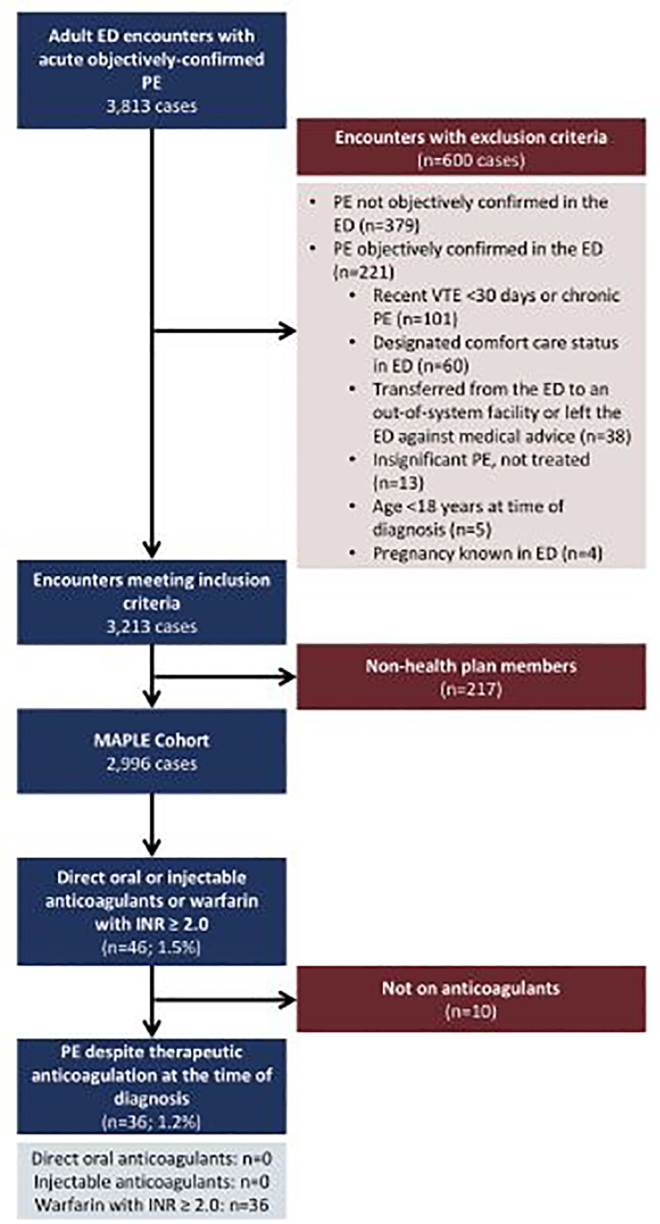
Cohort assembly of patients with breakthrough pulmonary embolism. *ED*, emergency department; *MAPLE*, Management of Acute PuLmonary Embolism; *INR*, international normalized ratio; *PE*, pulmonary embolism.

**Table 1:** Characteristics of patients with breakthrough pulmonary embolism (N=36).

Characteristics	N	%
Male	25	69
Age (years)		
30–44	8	22
45–64	8	22
>65	20	56
Indications for pre-arrival anticoagulation		
VTE treatment and prevention	21	58
Atrial fibrillation or flutter	13	36
Both	2	6
Active cancer	11	31

*VTE,* venous thromboembolism.

**Table 2:** Post-discharge changes in anticoagulation drug or dosing of patients with breakthrough pulmonary embolism (N=34).*

Change in anticoagulation drug or dosing	N	%
None	12	35
Discontinue warfarin (n=15)		
Start or continue enoxaparin	14	41
Start fondaparinux	1	3
Continue warfarin (n=7)		
Start enoxaparin	5	15
Increase warfarin dose	2	6
Inferior vena cava filter placement (n=4)		
Replace warfarin with enoxaparin	2	6
Supplement warfarin with enoxaparin	2	6

* Percentages do not add to 100% because patients who received inferior vena cava filters are included in the “discontinue warfarin” and “continue warfarin” subgroups.

**Table 3:** Characteristics of patients with breakthrough pulmonary embolism and subtherapeutic international normalized ratios (<2.0) in the 14 days preceding the index emergency department visit (N=15).

Characteristics	N	%
Age (years)		
30–44	3	20
45–64	2	13
>65	10	67
Change in anticoagulation drug or dosing		
None	9	60
Discontinue warfarin (n=4)		
Start enoxaparin	3	20
Continue enoxaparin	1	7
Continue warfarin (n=2)		
Start enoxaparin	1	7
Increase warfarin dose	1	7
